# RNA-seq analysis is easy as 1-2-3 with limma, Glimma and edgeR

**DOI:** 10.12688/f1000research.9005.3

**Published:** 2018-12-28

**Authors:** Charity W. Law, Monther Alhamdoosh, Shian Su, Xueyi Dong, Luyi Tian, Gordon K. Smyth, Matthew E. Ritchie

**Affiliations:** 1 The Walter and Eliza Hall Institute of Medical Research, Parkville, 3052, Australia; 2 Department of Medical Biology, The University of Melbourne, Parkville, 3010, Australia; 3 CSL Limited, Parkville, Victoria, 3010, Australia; 4 School of Mathematics and Statistics, The University of Melbourne, Parkville, 3010, Australia

**Keywords:** RNA sequencing, data analysis, gene expression

## Abstract

The ability to easily and efficiently analyse RNA-sequencing data is a key strength of the Bioconductor project. Starting with counts summarised at the gene-level, a typical analysis involves pre-processing, exploratory data analysis, differential expression testing and pathway analysis with the results obtained informing future experiments and validation studies. In this workflow article, we analyse RNA-sequencing data from the mouse mammary gland, demonstrating use of the popular **edgeR** package to import, organise, filter and normalise the data, followed by the **limma** package with its *voom* method, linear modelling and empirical Bayes moderation to assess differential expression and perform gene set testing. This pipeline is further enhanced by the **Glimma** package which enables interactive exploration of the results so that individual samples and genes can be examined by the user. The complete analysis offered by these three packages highlights the ease with which researchers can turn the raw counts from an RNA-sequencing experiment into biological insights using Bioconductor.

## Introduction

RNA-sequencing (RNA-seq) has become the primary technology used for gene expression profiling, with the genome-wide detection of differentially expressed genes between two or more conditions of interest one of the most commonly asked questions by researchers. The **edgeR** ^1^ and **limma** packages ^2^ available from the Bioconductor project ^3^ offer a well-developed suite of statistical methods for dealing with this question for RNA-seq data. In this article, we describe an **edgeR** - **limma** workflow for analysing RNA-seq data that takes gene-level counts as its input, and moves through pre-processing and exploratory data analysis before obtaining lists of differentially expressed (DE) genes and gene signatures. This analysis is enhanced through the use of interactive graphics from the **Glimma** package ^4^, that allows for a more detailed exploration of the data at both the sample and gene-level than is possible using static R plots.

The experiment analysed in this workflow is from Sheridan *et al.* (2015) ^5^ and consists of three cell populations (basal, luminal progenitor (LP) and mature luminal (ML)) sorted from the mammary glands of female virgin mice, each profiled in triplicate. RNA samples were sequenced across three batches on an Illumina HiSeq 2000 to obtain 100 base-pair single-end reads. The analysis outlined in this article assumes that reads obtained from an RNA-seq experiment have been aligned to an appropriate reference genome and summarised into counts associated with gene-specific regions. In this instance, reads were aligned to the mouse reference genome (mm10) using the R based pipeline available in the **Rsubread** package (specifically the align function ^6^ followed by featureCounts ^7^ for gene-level summarisation based on the in-built *mm10* RefSeq-based annotation). Count data for these samples can be downloaded from the Gene Expression Omnibus (GEO) http://www.ncbi.nlm.nih.gov/geo/ using GEO Series accession number GSE63310. Further information on experimental design and sample preparation is also available from GEO under this accession number.

## Data packaging

### Reading in count data

To get started with this analysis, download the file GSE63310_RAW.tar available online from http://www.ncbi.nlm.nih.gov/geo/download/?acc=GSE63310&format=file, and extract the relevant files from this archive. Each of these text files contains the raw gene-level counts for a given sample. Note that our analysis only includes the basal, LP and ML samples from this experiment (see associated file names below).

files <- c("GSM1545535_10_6_5_11.txt", "GSM1545536_9_6_5_11.txt", 
   "GSM1545538_purep53.txt", "GSM1545539_JMS8-2.txt", 
   "GSM1545540_JMS8-3.txt", "GSM1545541_JMS8-4.txt", 
   "GSM1545542_JMS8-5.txt", "GSM1545544_JMS9-P7c.txt", 
   "GSM1545545_JMS9-P8c.txt") 
read.delim(files[1], nrow=5)

##    EntrezID GeneLength Count 
## 1    497097       3634     1 
## 2 100503874       3259     0 
## 3 100038431       1634     0 
## 4     19888       9747     0 
## 5     20671       3130     1

Whilst each of the nine text files can be read into R separately and combined into a matrix of counts, **edgeR** offers a convenient way to do this in one step using the readDGE function. The resulting DGEList-object contains a matrix of counts with 27,179 rows associated with unique Entrez gene identifiers (IDs) and nine columns associated with the individual samples in the experiment.

library(limma) 
library(edgeR) 
x <- readDGE(files, columns=c(1,3)) 
class(x)

library(limma) 
library(edgeR) 
x <- readDGE(files, columns=c(1,3)) 
class(x)

dim(x)

## [1] 27179     9

If the counts from all samples were stored in a single file, the data can be read into R and then converted into a DGEList-object using the DGEList function.

### Organising sample information

For downstream analysis, sample-level information related to the experimental design needs to be associated with the columns of the counts matrix. This should include experimental variables, both biological and technical, that could have an effect on expression levels. Examples include cell type (basal, LP and ML in this experiment), genotype (wild-type, knock-out), phenotype (disease status, sex, age), sample treatment (drug, control) and batch information (date experiment was performed if samples were collected and analysed at distinct time points) to name just a few.

Our DGEList-object contains a samples data frame that stores both cell type (or group) and batch (sequencing lane) information, each of which consists of three distinct levels. Note that within x$ samples, library sizes are automatically calculated for each sample and normalisation factors are set to 1. For simplicity, we remove the GEO sample IDs (GSM*) from the column names of our DGEList-object x.

samplenames <- substring(colnames(x), 12, nchar(colnames(x))) 
samplenames

## [1] "10_6_5_11" "9_6_5_11"  "purep53"   "JMS8-2"    "JMS8-3"    "JMS8-4"    "JMS8-5" 
## [8] "JMS9-P7c"  "JMS9-P8c"

colnames(x) <- samplenames
group <- as.factor(c("LP", "ML", "Basal", "Basal", "ML", "LP", "Basal", "ML", "LP")) 
x$samples$group <- group 
lane <- as.factor(rep(c("L004","L006","L008"), c(3,4,2))) 
x$samples$lane <- lane 
x$samples

##                              files group lib.size norm.factors lane 
## 10_6_5_11 GSM1545535_10_6_5_11.txt    LP 32863052            1 L004 
## 9_6_5_11   GSM1545536_9_6_5_11.txt    ML 35335491            1 L004 
## purep53     GSM1545538_purep53.txt Basal 57160817            1 L004 
## JMS8-2       GSM1545539_JMS8-2.txt Basal 51368625            1 L006 
## JMS8-3       GSM1545540_JMS8-3.txt    ML 75795034            1 L006 
## JMS8-4       GSM1545541_JMS8-4.txt    LP 60517657            1 L006 
## JMS8-5       GSM1545542_JMS8-5.txt Basal 55086324            1 L006 
## JMS9-P7c   GSM1545544_JMS9-P7c.txt    ML 21311068            1 L008 
## JMS9-P8c   GSM1545545_JMS9-P8c.txt    LP 19958838            1 L008

### Organising gene annotations

A second data frame named genes in the DGEList-object is used to store gene-level information associated with rows of the counts matrix. This information can be retrieved using organism specific packages such as **Mus.musculus** ^8^ for mouse (or **Homo.sapiens** ^9^ for human) or the **biomaRt** package ^10, 11^ which interfaces the Ensembl genome databases in order to perform gene annotation. The type of information that can be retrieved includes gene symbols, gene names, chromosome names and locations, Entrez gene IDs, Refseq gene IDs and Ensembl gene IDs to name just a few. **biomaRt** primarily works off Ensembl gene IDs, whereas **Mus.musculus** packages information from various sources and allows users to choose between many different gene IDs as the key. The Entrez gene IDs available in our dataset were annotated using the **Mus.musculus** package to retrieve associated gene symbols and chromosome information.

library(Mus.musculus) 
geneid <- rownames(x) 
genes <- select(Mus.musculus, keys=geneid, columns=c("SYMBOL", "TXCHROM"), 
                keytype="ENTREZID")
dim(genes)

## [1] 27220     3

head(genes)

##      ENTREZID   SYMBOL   TXCHROM 
## 1      497097     Xkr4      chr1 
## 2   100503874  Gm19938      <NA> 
## 3   100038431  Gm10568      <NA> 
## 4       19888      Rp1      chr1 
## 5       20671    Sox17      chr1 
## 6       27395   Mrpl15      chr1

As with any gene ID, Entrez gene IDs may not map one-to-one to the gene information of interest. It is important to check for duplicated gene IDs and to understand the source of duplication before resolving them. Our gene annotation contains 28 genes that map to multiple chromosomes (e.g. gene Gm1987 is associated with “chr4” and “chr4_JH584294_random” and microRNA Mir5098 is associated with “chr2”, “chr5”, “chr8”, “chr11” and “chr17”). To resolve duplicate gene IDs one could combine all chromosome information from the multi-mapped genes, such that gene Gm1987 would be is assigned to “chr4 and chr4_JH584294_random”, or select one of the chromosomes to represent the gene with duplicate annotation. For simplicity we do the latter, keeping only the first occurrence of each gene ID.

genes <- genes[!duplicated(genes$ENTREZID),]

In this example, the gene order is the same in both the annotation and the data object. If this is not the case due to missing and/or rearranged gene IDs, the match function can be used to order genes correctly. The data frame of gene annotations is then added to the data object and neatly packaged in a DGEList-object containing raw count data with associated sample information and gene annotations.

x$genes <- genes
x

## An object of class "DGEList" 
## $samples 
##                              files group lib.size norm.factors lane 
## 10_6_5_11 GSM1545535_10_6_5_11.txt    LP 32863052            1 L004 
## 9_6_5_11   GSM1545536_9_6_5_11.txt    ML 35335491            1 L004 
## purep53     GSM1545538_purep53.txt Basal 57160817            1 L004 
## JMS8-2       GSM1545539_JMS8-2.txt Basal 51368625            1 L006 
## JMS8-3       GSM1545540_JMS8-3.txt    ML 75795034            1 L006 
## JMS8-4       GSM1545541_JMS8-4.txt    LP 60517657            1 L006 
## JMS8-5       GSM1545542_JMS8-5.txt Basal 55086324            1 L006 
## JMS9-P7c   GSM1545544_JMS9-P7c.txt    ML 21311068            1 L008 
## JMS9-P8c   GSM1545545_JMS9-P8c.txt    LP 19958838            1 L008 
## 
## $counts 
##            Samples 
## Tags        10_6_5_11 9_6_5_11 purep53 JMS8-2 JMS8-3 JMS8-4 JMS8-5 JMS9-P7c JMS9-P8c 
##   497097            1        2     342    526      3      3    535        2        0 
##   100503874         0        0       5      6      0      0      5        0        0 
##   100038431         0        0       0      0      0      0      1        0        0 
##   19888             0        1       0      0     17      2      0        1        0 
##   20671             1        1      76     40     33     14     98       18        8 
## 27174 more rows ... 
## 
## $genes 
##     ENTREZID  SYMBOL  TXCHROM 
## 1     497097    Xkr4     chr1 
## 2  100503874 Gm19938     <NA> 
## 3  100038431 Gm10568     <NA> 
## 4      19888     Rp1     chr1 
## 5      20671   Sox17     chr1 
## 27174 more rows ...

## Data pre-processing

### Transformations from the raw-scale

For differential expression and related analyses, gene expression is rarely considered at the level of raw counts since libraries sequenced at a greater depth will result in higher counts. Rather, it is common practice to transform raw counts onto a scale that accounts for such library size differences. Popular transformations include counts per million (CPM), log _2_-counts per million (log-CPM), reads per kilobase of transcript per million (RPKM), and fragments per kilobase of transcript per million (FPKM).

In our analyses, CPM and log-CPM transformations are used regularly although they do not account for gene length differences as RPKM and FPKM values do. Whilst RPKM and FPKM values can just as well be used, CPM and log-CPM values can be calculated using a counts matrix alone and will suffice for the type of comparisons we are interested in. Assuming that there are no differences in isoform usage between conditions, differential expression analyses look at gene expression changes between conditions rather than comparing expression across multiple genes or drawing conclusions on absolute levels of expression. In other words, gene lengths remain constant for comparisons of interest and any observed differences are a result of changes in condition rather than changes in gene length.

Here raw counts are converted to CPM and log-CPM values using the cpm function in **edgeR**. RPKM values are just as easily calculated as CPM values using the rpkm function in **edgeR** if gene lengths are available.

cpm <- cpm(x)
lcpm <- cpm(x, log=TRUE)


A CPM value of 1 for a gene equates to having 20 counts in the sample with the lowest sequencing depth (JMS9-P8c, library size *≈*20 million) or 76 counts in the sample with the greatest sequencing depth (JMS8-3, library size *≈*76 million).

The log-CPM values will be used for exploratory plots. When log=TRUE, the cpm function adds an offset to the CPM values before converting to the log2-scale. By default, the offset is 2/ *L* where 2 is the “prior count” and *L* is the average library size in millions, so the log-CPM values are related to the CPM values by log _2_(CPM + 2/ *L*). This calculation ensures that any two read counts with identical CPM values will also have identical log-CPM values. The prior count avoids taking the logarithm of zero, and also reduces spurious variability for genes with very low counts by shrinking all the inter-sample log-fold-changes towards zero, something that is helpful for exploratory plotting. For this dataset, the average library size is about 45.5 million, so *L ≈* 45.5 and the minimum log-CPM value for each sample becomes log _2_(2/45.5) = *−*4.51. In other words, a count of zero for this data maps to a log-CPM value of −4.51 after adding the prior count or offset:

L <- mean(x$samples$lib.size) * 1e-6
M <- median(x$samples$lib.size) * 1e-6
c(L, M)

## [1] 45.5 51.4

summary(lcpm)

##   10_6_5_11        9_6_5_11        purep53          JMS8-2          JMS8-3
## Min.   :-4.51   Min.   :-4.51   Min.   :-4.51   Min.   :-4.51   Min.   :-4.51
## 1st Qu.:-4.51   1st Qu.:-4.51   1st Qu.:-4.51   1st Qu.:-4.51   1st Qu.:-4.51
## Median :-0.68   Median :-0.36   Median :-0.10   Median :-0.09   Median :-0.43
## Mean   : 0.17   Mean   : 0.33   Mean   : 0.44   Mean   : 0.41   Mean   : 0.32
## 3rd Qu.: 4.29   3rd Qu.: 4.56   3rd Qu.: 4.60   3rd Qu.: 4.55   3rd Qu.: 4.58
## Max.   :14.76   Max.   :13.50   Max.   :12.96   Max.   :12.85   Max.   :12.96
##     JMS8-4          JMS8-5         JMS9-P7c        JMS9-P8c
## Min.   :-4.51   Min.   :-4.51   Min.   :-4.51   Min.   :-4.51
## 1st Qu.:-4.51   1st Qu.:-4.51   1st Qu.:-4.51   1st Qu.:-4.51
## Median :-0.41   Median :-0.07   Median :-0.17   Median :-0.33
## Mean   : 0.25   Mean   : 0.40   Mean   : 0.37   Mean   : 0.27
## 3rd Qu.: 4.32   3rd Qu.: 4.43   3rd Qu.: 4.60   3rd Qu.: 4.44
## Max.   :14.85   Max.   :13.19   Max.   :12.94   Max.   :14.01

Log-CPM values are also used in downstream linear modeling via **limma**’s voom function, although voom recomputes its own log-CPM values internally with a smaller prior count.

### Removing genes that are lowly expressed

All datasets will include a mix of genes that are expressed and those that are not expressed. Whilst it is of interest to examine genes that are expressed in one condition but not in another, some genes are unexpressed throughout all samples. In fact, 19% of genes in this dataset have zero counts across all nine samples.

table(rowSums(x$counts==0)==9)

## 
## FALSE  TRUE 
## 22026  5153

**Figure 1.**
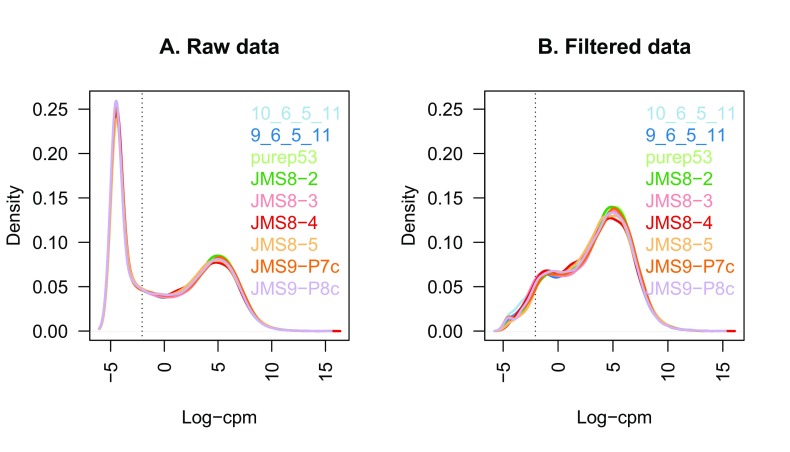
The density of log-CPM values for raw pre-filtered data ( **A**) and post-filtered data ( **B**) are shown for each sample. Dotted vertical lines mark the log-CPM threshold (equivalent to a CPM value of about 0.2) used in the filtering step.

Plotting the distribution log-CPM values shows that a sizeable proportion of genes within each sample are either unexpressed or lowly-expressed with log-CPM values that are small or negative ( Figure 1A).

Genes that do not have a worthwhile number of reads in any sample should be filtered out of the downstream analyses. There are several reasons for this. From a biological point of view, genes that not expressed at a biologically meaningful level in any condition are not of interest and are therefore best ignored. From a statistical point of view, removing low count genes allows the mean-variance relationship in the data to be estimated with greater reliability and also reduces the number of statistical tests that need to be carried out in downstream analyses looking at differential expression.

The filterByExpr function in the **edgeR** package provides an automatic way to filter genes, while keeping as many genes as possible with worthwhile counts.

keep.exprs <- filterByExpr(x, group=group)
x <- x[keep.exprs,, keep.lib.sizes=FALSE]
dim(x)

## [1] 16624     9



By default, the function keeps genes with about 10 read counts or more in a minimum number of samples, where the number of samples is chosen according to the minimum group sample size. The actual filtering uses CPM values rather than counts in order to avoid giving preference to samples with large library sizes. For this dataset, the median library size is about 51 million and 10/51 *≈* 0.2, so the filterByExpr function keeps genes that have a CPM of 0.2 or more in at least three samples. A biologically interesting gene should be expressed in at least three samples because all the cell type groups have three replicates. The cutoffs used depend on the sequencing depth and on the experimental design. If the library sizes had been larger then a lower CPM cutoff would have been chosen, because larger library sizes provide better resolution to explore more genes at lower expression levels. Alternatively, smaller library sizes decrease our ability to explore marginal genes and hence would have led to a higher CPM cutoff.

Using this criterion, the number of genes is reduced to 16,624, about 60% of the number that we started with. The log-CPM values after filtering show a nearly unimodel distribution for each sample ( Figure 1B). Note that subsetting the entire DGEList-object removes both the counts and the associated gene information for the filtered genes. The filtered DGEList-object keeps the gene information and the counts for the retained genes correctly associated.

Code to produce Figure 1 is given below:

lcpm.cutoff <- log2(10/M + 2/L)
library(RColorBrewer)
nsamples <- ncol(x)
col <- brewer.pal(nsamples, "Paired")
par(mfrow=c(1,2))
plot(density(lcpm[,1]), col=col[1], lwd=2, ylim=c(0,0.26), las=2, main="", xlab="")
title(main="A. Raw data", xlab="Log-cpm")
abline(v=lcpm.cutoff, lty=3)
for (i in 2:nsamples){
  den <- density(lcpm[,i])
  lines(den$x, den$y, col=col[i], lwd=2)
}
legend("topright", samplenames, text.col=col, bty="n")
lcpm <- cpm(x, log=TRUE)
plot(density(lcpm[,1]), col=col[1], lwd=2, ylim=c(0,0.26), las=2, main="", xlab="")
title(main="B. Filtered data", xlab="Log-cpm")
abline(v=lcpm.cutoff, lty=3)
for (i in 2:nsamples){
  den <- density(lcpm[,i])
  lines(den$x, den$y, col=col[i], lwd=2)
}
legend("topright", samplenames, text.col=col, bty="n")

### Normalising gene expression distributions

During the sample preparation or sequencing process, external factors that are not of biological interest can affect the expression of individual samples. For example, samples processed in the first batch of an experiment can have higher expression overall when compared to samples processed in a second batch. It is assumed that all samples should have a similar range and distribution of expression values. Normalisation is required to ensure that the expression distributions of each sample are similar across the entire experiment.

Any plot showing the per sample expression distributions, such as a density or boxplot, is useful in determining whether any samples are dissimilar to others. Distributions of log-CPM values are similar throughout all samples within this dataset ( Figure 1B).

Nonetheless, normalisation by the method of trimmed mean of M-values (TMM) ^12^ is performed using the calcNormFactors function in **edgeR**. The normalisation factors calculated here are used as a scaling factor for the library sizes. When working with DGEList-objects, these normalisation factors are automatically stored in x$samples$norm.factors. For this dataset the effect of TMM-normalisation is mild, as evident in the magnitude of the scaling factors, which are all relatively close to 1.

x <- calcNormFactors(x, method = "TMM")
x$samples$norm.factors

## [1] 0.894 1.025 1.046 1.046 1.016 0.922 0.996 1.086 0.984

To give a better visual representation of the effects of normalisation, the data was duplicated then adjusted so that the counts of the first sample are reduced to 5% of their original values, and in the second sample they are inflated to be 5-times larger.

x2 <- x
x2$samples$norm.factors <- 1
x2$counts[,1] <- ceiling(x2$counts[,1]*0.05)
x2$counts[,2] <- x2$counts[,2]*5

**Figure 2.**
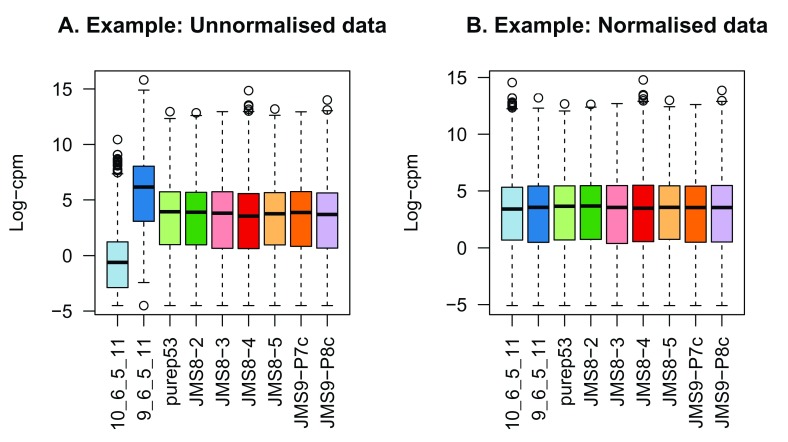
Example data: Boxplots of log-CPM values showing expression distributions for unnormalised data ( **A**) and normalised data ( **B**) for each sample in the modified dataset where the counts in samples 1 and 2 have been scaled to 5% and 500% of their original values respectively.

Figure 2 shows the expression distribution of samples for unnormalised and normalised data, where distributions are noticeably different pre-normalisation and are similar post-normalisation. Here the first sample has a small TMM scaling factor of 0.06, whereas the second sample has a large scaling factor of 6.08 – neither values are close to 1.

par(mfrow=c(1,2))
lcpm <- cpm(x2, log=TRUE)
boxplot(lcpm, las=2, col=col, main="")
title(main="A. Example: Unnormalised data", ylab="Log-cpm")
x2 <- calcNormFactors(x2)
x2$samples$norm.factors

## [1] 0.0577 6.0829 1.2202 1.1648 1.1966 1.0466 1.1505 1.2543 1.1090

lcpm <- cpm(x2, log=TRUE)
boxplot(lcpm, las=2, col=col, main="")
title(main="B. Example: Normalised data", ylab="Log-cpm")

### Unsupervised clustering of samples

In our opinion, one of the most important exploratory plots to examine for gene expression analyses is the multi-dimensional scaling (MDS) plot, or similar. The plot shows similarities and dissimilarities between samples in an unsupervised manner so that one can have an idea of the extent to which differential expression can be detected before carrying out formal tests. Ideally, samples would cluster well within the primary condition of interest, and any sample straying far from its group could be identified and followed up for sources of error or extra variation. If present, technical replicates should lie very close to one another.

Such a plot can be made in **limma** using the plotMDS function. The first dimension represents the leading-fold-change that best separates samples and explains the largest proportion of variation in the data, with subsequent dimensions having a smaller effect and being orthogonal to the ones before it. When experimental design involves multiple factors, it is recommended that each factor is examined over several dimensions. If samples cluster by a given factor in any of these dimensions, it suggests that the factor contributes to expression differences and is worth including in the linear modelling. On the other hand, factors that show little or no effect may be left out of downstream analysis.

**Figure 3.**
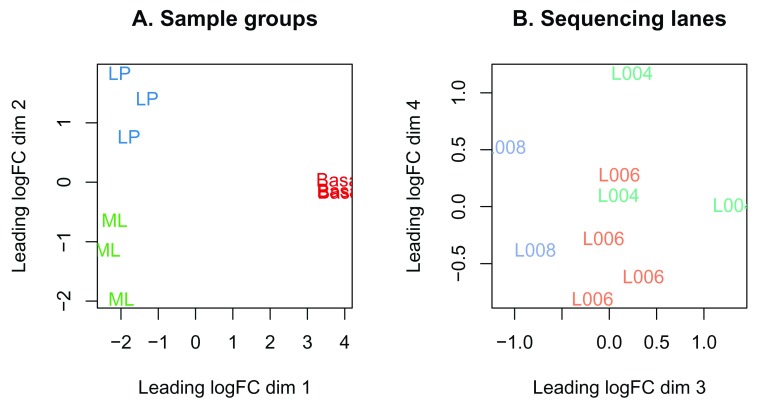
MDS plots of log-CPM values over dimensions 1 and 2 with samples coloured and labeled by sample groups ( **A**) and over dimensions 3 and 4 with samples coloured and labeled by sequencing lane ( **B**). Distances on the plot correspond to the leading fold-change, which is the average (root-mean-square) log _2_-fold-change for the 500 genes most divergent between each pair of samples by default.

In this dataset, samples can be seen to cluster well within experimental groups over dimension 1 and 2, and then separate by sequencing lane (sample batch) over dimension 3 ( Figure 3). Keeping in mind that the first dimension explains the largest proportion of variation in the data, notice that the range of values over the dimensions become smaller as we move to higher dimensions. Whilst all samples cluster by groups, the largest transcriptional difference is observed between basal and LP, and basal and ML over dimension 1. For this reason, it is expected that pairwise comparisons between cell populations will result in a greater number of DE genes for comparisons involving basal samples, and relatively small numbers of DE genes when comparing ML to LP. Datasets where samples do not cluster by experimental group may show little or no evidence of differential expression in the downstream analysis.

To create the MDS plots, we assign different colours to the factors of interest. Dimensions 1 and 2 are examined using the colour grouping defined by cell types.

lcpm <- cpm(x, log=TRUE)
par(mfrow=c(1,2))
col.group <- group
levels(col.group) <-  brewer.pal(nlevels(col.group), "Set1")
col.group <- as.character(col.group)
col.lane <- lane
levels(col.lane) <-  brewer.pal(nlevels(col.lane), "Set2")
col.lane <- as.character(col.lane)
plotMDS(lcpm, labels=group, col=col.group)
title(main="A. Sample groups")

Dimensions 3 and 4 are examined using the colour grouping defined by sequencing lanes (batch).

plotMDS(lcpm, labels=lane, col=col.lane, dim=c(3,4))
title(main="B. Sequencing lanes")

Alternatively, the **Glimma** package offers the convenience of an interactive MDS plot where multiple dimensions can be explored. The glMDSPlot function generates an html page (that is opened in a browser if launch=TRUE) with an MDS plot in the left panel and a barplot showing the proportion of variation explained by each dimension in the right panel. Clicking on the bars of the bar plot changes the pair of dimensions plotted in the MDS plot, and hovering over the individual points reveals the sample label. The colour scheme can be changed as well to highlight cell population or sequencing lane (batch). An interactive MDS plot of this dataset can be found at http://bioinf.wehi.edu.au/folders/limmaWorkflow/glimma-plots/MDS-Plot.html

library(Glimma)
glMDSPlot(lcpm, labels=paste(group, lane, sep="_"), groups=x$samples[,c(2,5)],
         launch=FALSE)

## Differential expression analysis

### Creating a design matrix and contrasts

In this study, it is of interest to see which genes are expressed at different levels between the three cell populations profiled. In our analysis, linear models are fitted to the data with the assumption that the underlying data is normally distributed. To get started, a design matrix is set up with both the cell population and sequencing lane (batch) information.

design <- model.matrix(^~^0+group+lane)
colnames(design) <- gsub("group", "", colnames(design))
design

##   Basal LP ML laneL006 laneL008
## 1     0  1  0        0        0
## 2     0  0  1        0        0
## 3     1  0  0        0        0
## 4     1  0  0        1        0
## 5     0  0  1        1        0
## 6     0  1  0        1        0
## 7     1  0  0        1        0
## 8     0  0  1        0        1
## 9     0  1  0        0        1
## attr(,"assign")
## [1] 1 1 1 2 2
## attr(,"contrasts")
## attr(,"contrasts")$group
## [1] "contr.treatment"
##
## attr(,"contrasts")$lane
## [1] "contr.treatment"

For a given experiment, there are usually several equivalent ways to set up an appropriate design matrix. For example, ~ 0+group+lane removes the intercept from the first factor, group, but an intercept remains in the second factor lane. Alternatively, ~ group+lane could be used to keep the intercepts in both group and lane. Understanding how to interpret the coefficients estimated in a given model is key here. We choose the first model for our analysis, as setting up model contrasts is more straight forward in the absence of an intercept for group. Contrasts for pairwise comparisons between cell populations are set up in **limma** using the makeContrasts function.

contr.matrix <- makeContrasts(
   BasalvsLP = Basal-LP,
   BasalvsML = Basal - ML, 
   LPvsML = LP - ML,
   levels = colnames(design))
contr.matrix

##           Contrasts
## Levels     BasalvsLP BasalvsML LPvsML
##   Basal            1         1      0
##   LP              -1         0      1
##   ML               0        -1     -1
##   laneL006         0         0      0
##   laneL008         0         0      0

A key strength of **limma**’s linear modelling approach is the ability accommodate arbitrary experimental complexity. Simple designs, such as the one in this workflow, with cell type and batch, through to more complicated factorial designs and models with interaction terms can be handled relatively easily. Where experimental or technical effects can be modelled using a random effect, another possibility in **limma** is to estimate correlations using duplicateCorrelation by specifying a block argument for both this function and in the lmFit linear modelling step.

### Removing heteroscedascity from count data

It has been shown that for RNA-seq count data, the variance is not independent of the mean ^13^ – this is true of raw counts or when transformed to log-CPM values. Methods that model counts using a Negative Binomial distribution assume a quadratic mean-variance relationship. In **limma**, linear modelling is carried out on the log-CPM values which are assumed to be normally distributed and the mean-variance relationship is accommodated using precision weights calculated by the voom function.

When operating on a DGEList-object, voom converts raw counts to log-CPM values by automatically extracting library sizes and normalisation factors from x itself. Additional normalisation to log-CPM values can be specified within voom using the normalize.method argument.

**Figure 4.**
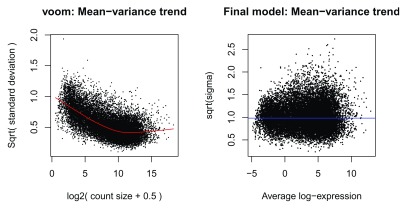
Means (x-axis) and variances (y-axis) of each gene are plotted to show the dependence between the two before voom is applied to the data ( **A**) and how the trend is removed after voom precision weights are applied to the data ( **B**). The plot on the left is created within the voom function which extracts residual variances from fitting linear models to log-CPM transformed data. Variances are then rescaled to quarter-root variances (or square-root of standard deviations) and plotted against the mean expression of each gene. The means are log _2_-transformed mean-counts with an offset of 2. The plot on the right is created using plotSA which plots log _2_ residual standard deviations against mean log-CPM values. The average log _2_ residual standard deviation is marked by a horizontal blue line. In both plots, each black dot represents a gene and a red curve is fitted to these points.

The mean-variance relationship of log-CPM values for this dataset is shown in Figure 4A. Typically, the “voom-plot” shows a decreasing trend between the means and variances resulting from a combination of technical variation in the sequencing experiment and biological variation amongst the replicate samples from different cell populations. Experiments with high biological variation usually result in flatter trends, where variance values plateau at high expression values. Experiments with low biological variation tend to result in sharp decreasing trends.

Moreover, the voom-plot provides a visual check on the level of filtering performed upstream. If filtering of lowly-expressed genes is insufficient, a drop in variance levels can be observed at the low end of the expression scale due to very small counts. If this is observed, one should return to the earlier filtering step and increase the expression threshold applied to the dataset.

Where sample-level variation is evident from earlier inspections of the MDS plot, the voomWithQualityWeights function can be used to simultaneously incorporate sample-level weights together with the abundance dependent weights estimated by voom ^14^. For an example of this approach, see Liu *et al.* (2016) ^15^.

v <- voom(x, design, plot=TRUE)
v

## An object of class "EList"
## $genes
##    ENTREZID SYMBOL TXCHROM
## 1    497097   Xkr4    chr1
## 5     20671  Sox17    chr1
## 6     27395 Mrpl15    chr1
## 7     18777 Lypla1    chr1
## 9     21399  Tcea1    chr1
## 16619 more rows ...
##
## $targets
##                              files group lib.size norm.factors lane
## 10_6_5_11 GSM1545535_10_6_5_11.txt    LP 29387429        0.894 L004
## 9_6_5_11   GSM1545536_9_6_5_11.txt    ML 36212498        1.025 L004
## purep53     GSM1545538_purep53.txt Basal 59771061        1.046 L004
## JMS8-2       GSM1545539_JMS8-2.txt Basal 53711278        1.046 L006
## JMS8-3       GSM1545540_JMS8-3.txt    ML 77015912        1.016 L006
## JMS8-4       GSM1545541_JMS8-4.txt    LP 55769890        0.922 L006
## JMS8-5       GSM1545542_JMS8-5.txt Basal 54863512        0.996 L006
## JMS9-P7c   GSM1545544_JMS9-P7c.txt    ML 23139691        1.086 L008
## JMS9-P8c   GSM1545545_JMS9-P8c.txt    LP 19634459        0.984 L008
##
## $E
##         Samples
## Tags     10_6_5_11 9_6_5_11 purep53 JMS8-2 JMS8-3 JMS8-4 JMS8-5 JMS9-P7c JMS9-P8c
##   497097     -4.29    -3.86   2.519  3.293  -4.46  -3.99  3.287   -3.210    -5.30
##   20671      -4.29    -4.59   0.356 -0.407  -1.20  -1.94  0.844   -0.323    -1.21
##   27395       3.88     4.41   4.517  4.562   4.34   3.79  3.899    4.340     4.12
##   18777       4.71     5.57   5.396  5.162   5.65   5.08  5.060    5.751     5.14
##   21399       4.79     4.75   5.370  5.122   4.87   4.94  5.138    5.031     4.98
## 16619 more rows ...
##
## $weights
##       [,1]  [,2]  [,3]  [,4]  [,5]  [,6]  [,7]  [,8]  [,9]
## [1,]  1.08  1.33  19.8 20.27  1.99  1.40 20.49  1.11  1.08
## [2,]  1.17  1.46   4.8  8.66  3.61  2.63  8.76  3.21  2.54
## [3,] 20.22 25.57  30.4 28.53 31.35 25.74 28.72 21.20 16.66
## [4,] 26.95 32.51  33.6 33.23 34.23 32.35 33.33 30.35 24.26
## [5,] 26.61 28.50  33.6 33.21 33.57 32.00 33.31 25.17 23.57
## 16619 more rows ...
##
## $design
##   Basal LP ML laneL006 laneL008
## 1     0  1  0        0        0
## 2     0  0  1        0        0
## 3     1  0  0        0        0
## 4     1  0  0        1        0
## 5     0  0  1        1        0
## 6     0  1  0        1        0
## 7     1  0  0        1        0
## 8     0  0  1        0        1
## 9     0  1  0        0        1
## attr(,"assign")
## [1] 1 1 1 2 2
## attr(,"contrasts")
## attr(,"contrasts")$group
## [1] "contr.treatment"
##
## attr(,"contrasts")$lane
## [1] "contr.treatment"


Note that the other data frames stored within the DGEList-object that contain gene- and sample-level information, are retained in the EList-object v created by voom. The v$genes data frame is equivalent to x$genes, v$targets is equivalent to x$samples, and the expression values stored in v$E is analogous to x$counts, albeit on a transformed scale. In addition to this, the voom EList-object has a matrix of precision weights v$weights and stores the design matrix in v$design.

### Fitting linear models for comparisons of interest

Linear modelling in **limma** is carried out using the lmFit and contrasts.fit functions originally written for application to microarrays. The functions can be used for both microarray and RNA-seq data and fit a separate model to the expression values for each gene. Next, empirical Bayes moderation is carried out by borrowing information across all genes to obtain more precise estimates of gene-wise variability ^16^. The model’s residual variances are plotted against average expression values in Figure 4B. It can be seen from this plot that the variance is no longer dependent on the mean expression level.

vfit <- lmFit(v, design)
vfit <- contrasts.fit(vfit, contrasts=contr.matrix)
efit <- eBayes(vfit)
plotSA(efit)

### Examining the number of DE genes

For a quick look at differential expression levels, the number of significantly up- and down-regulated genes can be summarised in a table. Significance is defined using an adjusted *p*-value cutoff that is set at 5% by default. For the comparison between expression levels in basal and LP, 4,648 genes are found to be down-regulated in basal relative to LP and 4,863 genes are up-regulated in basal relative to LP – a total of 9,511 DE genes. A total of 9,598 DE genes are found between basal and ML (4,927 down- and 4,671 up-regulated genes), and a total of 5,652 DE genes are found between LP and ML (3,135 down- and 2,517 up-regulated). The larger numbers of DE genes observed for comparisons involving the basal population are consistent with our observations from the MDS plots.

summary(decideTests(efit))

##        BasalvsLP BasalvsML LPvsML
## Down        4648      4927   3135
## NotSig      7113      7026  10972
## Up          4863      4671   2517

Some studies require more than an adjusted *p*-value cutoff. For a stricter definition on significance, one may require log-fold-changes (log-FCs) to be above a minimum value. The *treat* method ^17^ can be used to calculate *p*-values from empirical Bayes moderated *t*-statistics with a minimum log-FC requirement. The number of differentially expressed genes are reduced to a total of 3,648 DE genes for basal versus LP, 3,834 DE genes for basal versus ML, and 414 DE genes for LP versus ML when testing requires genes to have a log-FC that is significantly greater than 1 (equivalent to a 2-fold difference between cell types on the original scale).

tfit <- treat(vfit, lfc=1)
dt <- decideTests(tfit)
summary(dt)

##         BasalvsLP BasalvsML LPvsML
## Down         1632      1777    224
## NotSig      12976     12790  16210
## Up           2016      2057    190

**Figure 5.**
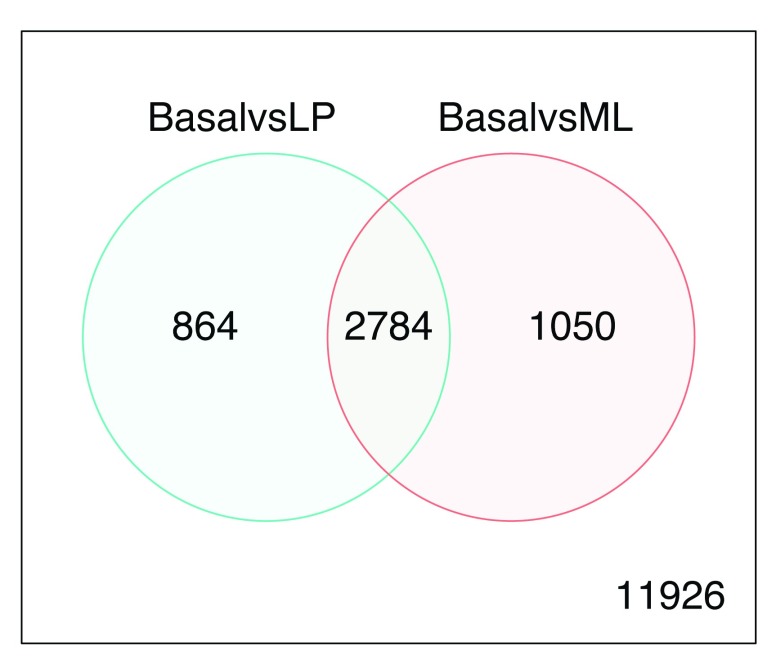
Venn diagram showing the number of genes DE in the comparison between basal versus LP only (left), basal versus ML only (right), and the number of genes that are DE in both comparisons (center). The number of genes that are not DE in either comparison are marked in the bottom-right.

Genes that are DE in multiple comparisons can be extracted using the results from decideTests, where 0s represent genes that are not DE, 1s represent genes that are up-regulated, and -1s represent genes that are down-regulated. A total of 2,784 genes are DE in both basal versus LP and basal versus ML ( Figure 5), twenty of which are listed below. The write.fit function can be used to extract and write results for all three comparisons to a single output file.

de.common <- which(dt[,1]!=0 & dt[,2]!=0)
length(de.common)

## [1] 2784

head(tfit$genes$SYMBOL[de.common], n=20)

##  [1] "Xkr4"          "Rgs20"         "Cpa6"           "A830018L16Rik" "Sulf1"
##  [6] "Eya1"          "Msc"           "Sbspon"         "Pi15"          "Crispld1"
## [11] "Kcnq5"         "Rims1"         "Khdrbs2"        "Ptpn18"        "Prss39"
## [16] "Arhgef4"       "Cnga3"         "2010300C02Rik"  "Aff3"          "Npas2"

vennDiagram(dt[,1:2], circle.col=c("turquoise", "salmon"))
write.fit(tfit, dt, file="results.txt")

### Examining individual DE genes from top to bottom

The top DE genes can be listed using topTreat for results using treat (or topTable for results using eBayes). By default topTreat arranges genes from smallest to largest adjusted *p*-value with associated gene information, log-FC, average log-CPM, moderated *t*-statistic, raw and adjusted *p*-value for each gene. The number of top genes displayed can be specified, where n=Inf includes all genes. Genes *Cldn7* and *Rasef* are amongst the top DE genes for both basal versus LP and basal versus ML.

basal.vs.lp <- topTreat(tfit, coef=1, n=Inf)
basal.vs.ml <- topTreat(tfit, coef=2, n=Inf)
head(basal.vs.lp)

##        ENTREZID SYMBOL TXCHROM logFC AveExpr     t  P.Value adj.P.Val
## 12759     12759    Clu   chr14 -5.46    8.86 -33.6 1.72e-10  1.71e-06
## 53624     53624  Cldn7   chr11 -5.53    6.30 -32.0 2.58e-10  1.71e-06
## 242505   242505  Rasef    chr4 -5.94    5.12 -31.3 3.08e-10  1.71e-06
## 67451     67451   Pkp2   chr16 -5.74    4.42 -29.9 4.58e-10  1.74e-06
## 228543   228543   Rhov    chr2 -6.26    5.49 -29.1 5.78e-10  1.74e-06
## 70350     70350  Basp1   chr15 -6.08    5.25 -28.3 7.27e-10  1.74e-06

head(basal.vs.ml)

##        ENTREZID  SYMBOL TXCHROM logFC AveExpr     t  P.Value adj.P.Val
## 242505   242505   Rasef    chr4 -6.53    5.12 -35.1 1.23e-10  1.24e-06
## 53624     53624   Cldn7   chr11 -5.50    6.30 -31.7 2.77e-10  1.24e-06
## 12521     12521    Cd82    chr2 -4.69    7.07 -31.4 2.91e-10  1.24e-06
## 20661     20661   Sort1    chr3 -4.93    6.70 -30.7 3.56e-10  1.24e-06
## 71740     71740 Nectin4    chr1 -5.58    5.16 -30.6 3.72e-10  1.24e-06
## 12759     12759     Clu   chr14 -4.69    8.86 -28.0 7.69e-10  1.48e-06

### Useful graphical representations of differential expression results

To summarise results for all genes visually, mean-difference plots, which display log-FCs from the linear model fit against the average log-CPM values can be generated using the plotMD function, with the differentially expressed genes highlighted.

plotMD(tfit, column=1, status=dt[,1], main=colnames(tfit)[1], xlim=c(-8,13))

**Figure 6.**
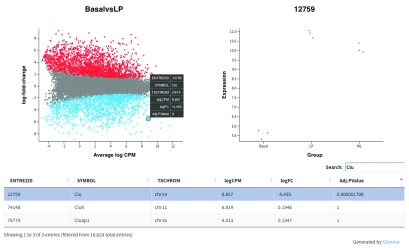
Interactive mean-difference plot generated using Glimma. Summary data (log-FCs versus log-CPM values) are shown in the left panel which is linked to the individual values per sample for a selected gene in the right panel. A table of results is also displayed below these figures, along with a search bar to allow users to look up a particular gene using the annotation information available, e.g. the Gene symbol identifier *Clu*.

**Glimma** extends this functionality by providing an interactive mean-difference plot via the glMDPlot function. The output of this function is an html page, with summarised results in the left panel (similar to what is output by plotMD), and the log-CPM values from individual samples for a selected gene in the right panel, with a table of results below the plots ( Figure 6). This interactive display allows the user to search for particular genes based on the annotation provided (e.g. Gene symbol identifier), which is not possible in a static R plot.

glMDPlot(tfit, coef=1, status=dt, main=colnames(tfit)[1],
         side.main="ENTREZID", counts=lcpm, groups=group, launch=FALSE)

The mean-difference plot generated by the command above is available online (see http://bioinf.wehi.edu.au/folders/limmaWorkflow/glimma-plots/MD-Plot.html). The interactivity provided by the **Glimma** package allows additional information to be presented in a single graphical window. **Glimma** is implemented in R and Javascript, with the R code generating the data which is converted into graphics using the Javascript library D3 ( https://d3js.org), with the Bootstrap library handling layouts and Datatables generating the interactive searchable tables. This allows plots to be viewed in any modern browser, which is convenient for including them as linked files from an Rmarkdown report of the analysis.

Plots shown previously include either all of the genes that are expressed in any one condition (such as the Venn diagram of common DE genes or mean-difference plot) or look at genes individually (log-CPM values shown in right panel of the interactive mean-difference plot). Heatmaps allow users to look at the expression of a subset of genes. This can give useful insight into the expression of individual groups and samples without losing perspective of the overall study when focusing on individual genes, or losing resolution when examining patterns averaged over thousands of genes at the same time.

**Figure 7.**
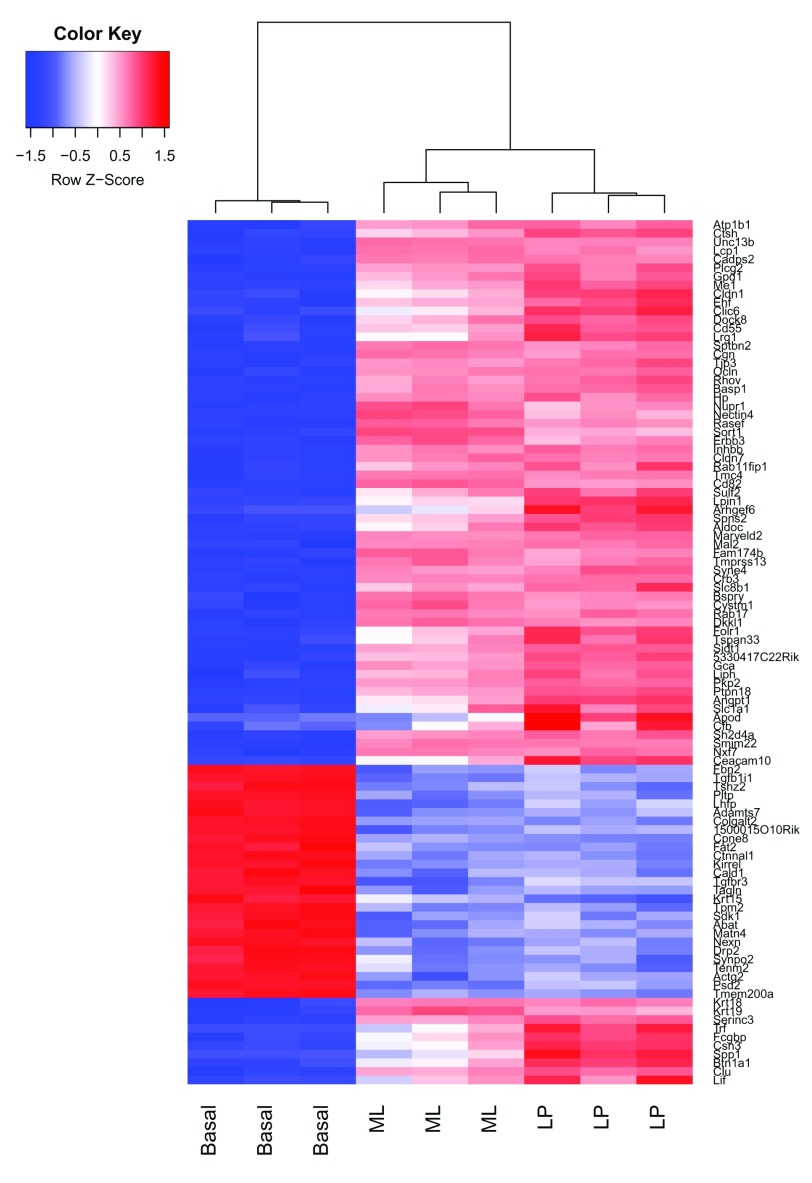
Heatmap of log-CPM values for top 100 genes DE in basal versus LP. Expression across each gene (or row) have been scaled so that mean expression is zero and standard deviation is one. Samples with relatively high expression of a given gene are marked in red and samples with relatively low expression are marked in blue. Lighter shades and white represent genes with intermediate expression levels. Samples and genes have been reordered by the method of hierarchical clustering. A dendrogram is shown for the sample clustering.

A heatmap is created for the top 100 DE genes (as ranked by adjusted *p*-value) from the basal versus LP contrast using the heatmap.2 function from the **gplots** package ( Figure 7). The heatmap correctly clusters samples by cell type and reorders the genes into blocks with similar expression patterns. From the heatmap, we observe that the expression of ML and LP samples are very similar for the top 100 DE genes between basal and LP.

library(gplots)
basal.vs.lp.topgenes <- basal.vs.lp$ENTREZID[1:100]
i <- which(v$genes$ENTREZID %in% basal.vs.lp.topgenes)
mycol <- colorpanel(1000,"blue","white","red")

heatmap.2(lcpm[i,], scale="row",
   labRow=v$genes$SYMBOL[i], labCol=group,
   col=mycol, trace="none", density.info="none", 
   margin=c(8,6), lhei=c(2,10), dendrogram="column")

## Gene set testing with camera

We finish off this analysis with some gene set testing by applying the *camera* method ^18^ to the *c2* gene signatures from the Broad Institute’s MSigDB c2 collection ^19^ that have been adapted for mouse and are available as Rdata objects from http://bioinf.wehi.edu.au/software/MSigDB/. Other useful gene sets derived from MSigDB for both human and mouse, such as the hallmark gene sets, are also available from this site. C2 gene sets have been curated from online databases, publications and domain experts, and hallmark gene sets are selected from MSigDB to have well-defined biological states or processes.

load(url("http://bioinf.wehi.edu.au/software/MSigDB/mouse_c2_v5p1.rdata")) 
idx <- ids2indices(Mm.c2,id=rownames(v)) 
cam.BasalvsLP <- camera(v,idx,design,contrast=contr.matrix[,1]) 
head(cam.BasalvsLP,5)

##                                             NGenes Direction   PValue      FDR 
## LIM_MAMMARY_STEM_CELL_UP                       791        Up 1.77e-18 8.36e-15 
## LIM_MAMMARY_STEM_CELL_DN                       683      Down 4.03e-14 8.69e-11 
## ROSTY_CERVICAL_CANCER_PROLIFERATION_CLUSTER    170        Up 5.52e-14 8.69e-11 
## LIM_MAMMARY_LUMINAL_PROGENITOR_UP	           94	   Down 2.74e-13 3.23e-10 
## SOTIRIOU_BREAST_CANCER_GRADE_1_VS_3_UP         190        Up 5.16e-13 4.87e-10

cam.BasalvsML <- camera(v,idx,design,contrast=contr.matrix[,2]) 
head(cam.BasalvsML,5)

##                                     NGenes Direction   PValue      FDR 
## LIM_MAMMARY_STEM_CELL_UP               791        Up 1.68e-22 7.92e-19 
## LIM_MAMMARY_STEM_CELL_DN               683      Down 7.79e-18 1.84e-14 
## LIM_MAMMARY_LUMINAL_MATURE_DN          172        Up 9.74e-16 1.53e-12 
## LIM_MAMMARY_LUMINAL_MATURE_UP          204      Down 1.15e-12 1.36e-09 
## NAKAYAMA_SOFT_TISSUE_TUMORS_PCA2_UP    137        Up 2.24e-12 1.88e-09

cam.LPvsML <- camera(v,idx,design,contrast=contr.matrix[,3]) 
head(cam.LPvsML,5)

##                                          NGenes Direction   PValue      FDR 
## LIM_MAMMARY_LUMINAL_MATURE_DN               172        UP 6.73e-14 2.35e-10 
## LIM_MAMMARY_LUMINAL_MATURE_UP               204      Down 9.97e-14 2.35e-10 
## LIM_MAMMARY_LUMINAL_PROGENITOR_UP            94        Up 1.32e-11 2.08e-08 
## REACTOME_RESPIRATORY_ELECTRON_TRANSPORT      94      Down 7.01e-09 8.28e-06 
## REACTOME_RNA_POL_I_PROMOTER_OPENING          46      Down 2.04e-08 1.93e-05

The camera function performs a competitive test to assess whether the genes in a given set are highly ranked in terms of differential expression relative to genes that are not in the set. It uses **limma**’s linear model framework, taking both the design matrix and contrast matrix (if present) and accommodates the observational-level weights from *voom* in the testing procedure. After adjusting the variance of the resulting gene set test statistic by a variance inflation factor that depends on the gene-wise correlation (which is set to 0.01 by default, but can be estimated from the data) and the size of the set, a *p*-value is returned and adjusted for multiple testing.

This experiment is the RNA-seq equivalent of the dataset generated by Lim *et al.* (2010) ^20^, who used Illumina microarrays to profile the same sorted cell populations, so it is reassuring to see the gene signatures from this earlier publication coming up at the top of the list for each contrast. We make a barcodeplot of the Lim *et al.* (2010) Mature Luminal gene sets (Up and Down) in the LP versus ML contrast. Note that these sets go in the opposite direction in our dataset due to our parameterization which compares LP against ML rather than the other way around (if the contrast were reversed, the directions would be consistent).

barcodeplot(efit$t[,3], index=idx$LIM_MAMMARY_LUMINAL_MATURE_UP, 
            index2=idx$LIM_MAMMARY_LUMINAL_MATURE_DN, main="LPvsML")

**Figure 8.**
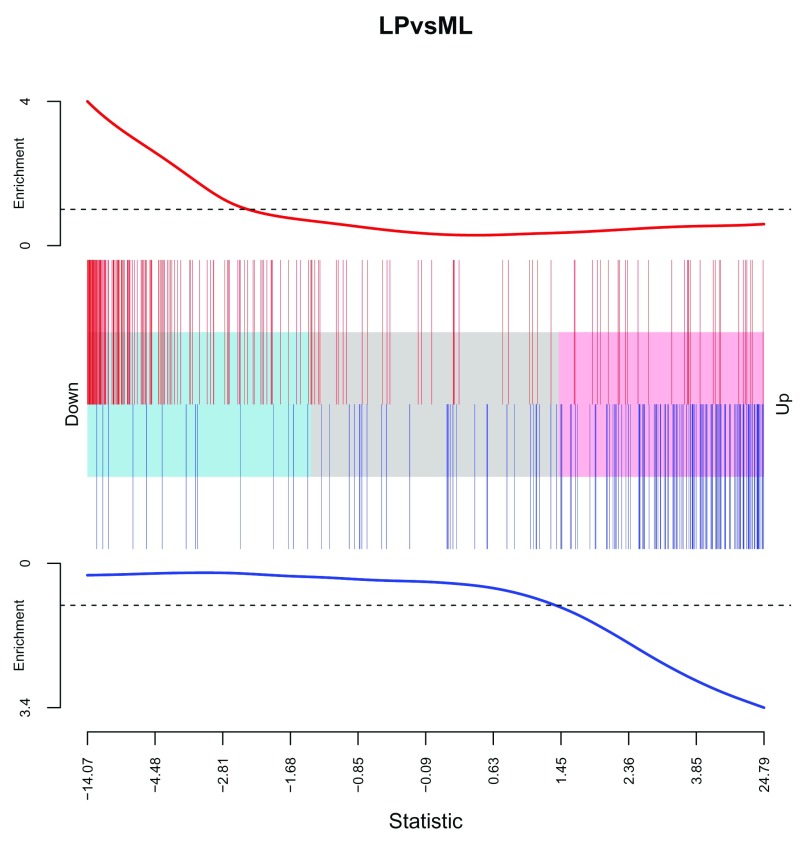
Barcode plot of LIM_MAMMARY_LUMINAL_MATURE_UP (red bars, top of plot) and LIM_MAMMARY_LUMINAL_MATURE_DN (blue bars, bottom of plot) gene sets in the LP versus ML contrast. For each set, an enrichment line that shows the relative enrichment of the vertical bars in each part of the plot is displayed. The experiment of Lim *et al.* (2010) is very similar to the current one, with the same sorting strategy used to obtain the different cell populations, except that microarrays were used instead of RNA-seq to profile gene expression. Note that the inverse correlation (the up gene set is down and the down gene set is up) is a result of the way the contrast has been set up (LP versus ML) – if reversed, the directionality would agree.

Other gene set tests are available in **limma**, such as the self-contained tests by *mroast* ^21^. Whilst *camera* is ideal for testing a large database of gene sets and observing which of them rank highly relative to others (as shown above), self-contained tests are better for focused testing of one or a few specifically chosen sets to see if they are DE in their own right. In other words, *camera* is more appropriate when “fishing” for gene sets of interest, whereas *mroast* tests sets that are already of interest for significance.

## Software availability

This RNA-seq workflow makes use of various packages available from version 3.8 of the Bioconductor project, running on R ^22^ version 3.5.1. Besides the software highlighted in this article ( **limma**, **Glimma** and **edgeR**) it requires a number of other packages, including **gplots** ^23^ and **RColorBrewer** and the gene annotation package **Mus.musculus**. This document was compiled using **knitr** ^24– 26^. Version numbers for all packages used are shown below. The **RNAseq123** Bioconductor workflow package available from https://bioconductor.org/packages/RNAseq123 contains both an English and Chinese (Mandarin) vignette of this article along with code to perform the complete analysis. Installation of this package manages all of the above-mentioned dependencies and is a useful resource for delivering hands-on training on RNA-seq data analysis.

sessionInfo()

## R version 3.5.1 (2018-07-02) 
## Platform: x86_64-apple-darwin15.6.0 (64-bit) 
## Running under: macOS High Sierra 10.13.6 
## 
## Matrix products: default
## BLAS: /Library/Frameworks/R.framework/Versions/3.5/Resources/lib/libRblas.0.dylib
## LAPACK: /Library/Frameworks/R.framework/Versions/3.5/Resources/lib/libRlapack.dylib
## 
## locale: 
## [1] en_AU.UTF-8/en_AU.UTF-8/en_AU.UTF-8/C/en_AU.UTF-8/en_AU.UTF-8 
## 
## attached base packages: 
## [1] parallel  stats4     stats     graphics grDevices utils     datasets methods 
## [9] base 
## 
## other attached packages: 
##  [1] gplots_3.0.1                             RColorBrewer_1.1-2 
##  [3] Mus.musculus_1.3.1                       TxDb.Mmusculus.UCSC.mm10.knownGene_3.4.4 
##  [5] org.Mm.eg.db_3.7.0                       GO.db_3.7.0 
##  [7] OrganismDbi_1.24.0                       GenomicFeatures_1.34.1 
##  [9] GenomicRanges_1.34.0                     GenomeInfoDb_1.18.1 
## [11] AnnotationDbi_1.44.0                     IRanges_2.16.0 
## [13] S4Vectors_0.20.1                         Biobase_2.42.0 
## [15] BiocGenerics_0.28.0                      edgeR_3.24.0 
## [17] Glimma_1.10.0                            limma_3.38.3 
## [19] knitr_1.20                               BiocStyle_2.10.0 
## 
## loaded via a namespace (and not attached):
##  [1] httr_1.3.1                  bit64_0.9-7                 jsonlite_1.5 
##  [4] R.utils_2.7.0               gtools_3.8.1                assertthat_0.2.0 
##  [7] BiocManager_1.30.4          highr_0.7                   RBGL_1.58.1 
## [10] blob_1.1.1                  GenomeInfoDbData_1.2.0      Rsamtools_1.34.0 
## [13] yaml_2.2.0                  progress_1.2.0              RSQLite_2.1.1 
## [16] backports_1.1.2             lattice_0.20-38             digest_0.6.18 
## [19] XVector_0.22.0              htmltools_0.3.6             Matrix_1.2-15 
## [22] R.oo_1.22.0                 XML_3.98-1.16               pkgconfig_2.0.2 
## [25] biomaRt_2.38.0              zlibbioc_1.28.0             gdata_2.18.0 
## [28] BiocParallel_1.16.2         SummarizedExperiment_1.12.0 magrittr_1.5 
## [31] crayon_1.3.4                memoise_1.1.0               evaluate_0.12 
## [34] R.methodsS3_1.7.1           graph_1.60.0                tools_3.5.1 
## [37] prettyunits_1.0.2           hms_0.4.2                   matrixStats_0.54.0 
## [40] stringr_1.3.1               locfit_1.5-9.1              DelayedArray_0.8.0 
## [43] Biostrings_2.50.1           compiler_3.5.1              caTools_1.17.1.1
## [46] rlang_0.3.0.1               grid_3.5.1                  RCurl_1.95-4.11 
## [49] bitops_1.0-6                rmarkdown_1.10              DBI_1.0.0 
## [52] R6_2.3.0                    GenomicAlignments_1.18.0    rtracklayer_1.42.1 
## [55] bit_1.1-14                  rprojroot_1.3-2             KernSmooth_2.23-15 
## [58] stringi_1.2.4               Rcpp_1.0.0
