# Supplementary material for: RNA-seq analysis is easy as 1-2-3 with limma, Glimma and edgeR
Source: F1000Res. 2018 Dec 28;5:ISCB Comm J-1408. Originally published 2016 Jun 17. [Version 3] doi: 10.12688/f1000research.9005.3 (PMC4937821; doi:10.12688/f1000research.9005.3)
Supplement: Peer review history

# Peer review: Referee response for version 1

**DOI:** 10.5256/f1000research.9688.r14437

**Published:** 2016-07-06

**Authors:** Jovana Maksimovic

**Affiliations:** 1 Bioinformatics research group, Murdoch Childrens Research Institute, Murdoch Children's Research Institute, Parkville, Vic, Australia

This workflow outlines a step-by-step differential expression analysis of a publicly available RNA-seq dataset using several well-established Bioconductor packages such as limma and edgeR and the novel Glimma package. The workflow describes in detail the key steps that are generally performed as part of most standard, count-based RNAseq analyses: pre-processing, exploratory data analysis, differential expression testing and pathway analysis. Each of the steps is carefully explained and broken down into a series of logical substeps, which also include R code and example output. Furthermore, this workflow is a good example of how multiple Bioconductor packages can be applied in a real-world RNAseq analysis.  

 Overall, I think this is a very clearly written and well-explained workflow that not only highlights the utility of combining Bioconductor packages for RNAseq analysis but also demystifies the process for those who may be interested in learning what an RNAseq analysis involves. Although some familiarity with R and statistical concepts would be advantageous, they are not necessary to be able to follow the logic behind the steps and what each step is trying to achieve.

 I have tested the R code under R 3.3.0 and Bioconductor 3.0 and it ran without errors.

**Minor comments:**

- As this workflow will be of particular use for those who are very new to RNAseq analysis, I think it should be explained why 1 CPM is chosen as the cutoff for lowly expressed genes. In addition, it may be useful to show the relationship between CPM and raw counts and how the selected cutoff relates to numbers of reads.
- In the gene set testing section, it is worth mentioning that the Hallmark gene sets (also available from the Broad MSigDB) may be a good starting point for pathway analysis as they “summarise and represent specific well-defined, states or processes”.
- Also, for those who are new to RNAseq analysis, it may be worth mentioning that there are other gene set testing methods available in limma e.g. roast etc. and a very brief explanation of when the different methods are appropriate.   
- General comment regarding Glimma plots: it would be handy to see the gene symbol (if available) as well as EntrezID in the title of the scatter plot next to the MD plot.

I have read this submission. I believe that I have an appropriate level of expertise to confirm that it is of an acceptable scientific standard.

# Response

**Published:** 2016-11-30

**Authors:** Charity Law

**Affiliations:** The Walter and Eliza Hall Institute of Medical Research, Australia

Dear Jovana, thank-you for the helpful suggestions. We have added extra text to explain why a CPM value of 1 was chosen and how this value relates to raw counts. We have also given more detail on c2 and Hallmark gene sets, as well as mentioning roast and when one might chose to use roast over camera.

# Peer review: Referee response for version 1

**DOI:** 10.5256/f1000research.9688.r14439

**Published:** 2016-07-06

**Authors:** Maria A. Doyle

**Affiliations:** 1 Research Computing Facility, Peter MacCallum Cancer Centre, Melbourne, Vic, Australia

**Editorial Note from F1000Research - 7th July 2016:**

****

**the status of this review was changed after publication from "Approved with Reservations" to "Approved". The referee's reservation was caused by using the current release (Bioconductor 3.3) as opposed to the development release (Bioconductor 3.4) as used in the article.**

****

**The following text has been removed:**

****

**My only reservation to approving the article is that I encountered these two errors when running the Glimma code in the article. I used R 3.3.0 and the most recent version of Glimma from Bioconductor (Glimma 1.0.0) but the sessionInfo() in the article shows Glimma 1.1.1 was used which may account for the errors:**

****

**1) With this line of code no plot was produced and it gave the error below:**

**glMDSPlot(lcpm, labels=paste(group, lane, sep="_"),**

**+           groups=x$samples[,c(2,5)], launch=TRUE)**

**Error in glScatter.default(points, xval = "dim1", yval = "dim2", xlab = "Dimension 1",  :**

**group does not correspond to a column**

****

**2) With this line of code the table (shown in Fig 6.) didn't appear, just the 2 plots:**

**glMDPlot(tfit, coef=1, status=dt[,1], main=colnames(tfit)[1],**

**counts=x$counts, samples=colnames(x), anno=x$genes, groups=group,**

**id.column="ENTREZID", display.columns=c("SYMBOL", "ENTREZID"),**

**search.by="SYMBOL", launch=FALSE)**

 This article is a very nice description (nicely written, nicely explained) of an RNA-seq workflow, starting from gene counts and proceeding through a standard differential expression analysis, using 3 complementary Bioconductor packages edgeR, limma & Glimma. It explains clearly how to perform the steps of a differential expression analysis and also how to generate useful plots for visualising the data e.g. MDSplot, MDplot, barcodeplot, heatmap etc.

 Two of the packages, edgeR and limma, are well established while Glimma is the new kid on the block. It's a tool along the lines of the web-based Degust (http://www.vicbioinformatics.com/degust/), in that it enables interactive exploration of RNA-seq data. It would seem to be a very useful addition to R RNA-seq workflows as the interactive html plots it can generate (MDS, MD etc) make exploring the results easier (especially by non-R savvy collaborators) and it could help save an analyst's time and effort through not having to reproduce static plots e.g. to highlight different genes. With the Glimma MDplot I like that you can search for a gene and see a plot of the log-cpm counts for that gene in the samples.

 It was great to be able to try out the workflow really easily by downloading the data file linked to in the paper (from GEO), with no processing required other than unzipping the files, and the code in the article all worked, giving the same results shown in the paper with the exception of two errors described below.

 Minor comments:

 The article says the workflow is available from Bioconductor here http://www.bioconductor.org/help/workflows/ but it doesn't seem to be there, however it is available at the other location mentioned http://bioinf.wehi.edu.au/folders/limmaWorkflow/

 The section title "Removing genes that are not expressed" suggests only genes with no expression are removed, however the paragraph then explains that genes lowly expressed are also removed so for greater clarity maybe that could be changed to something like "Removing genes that are not sufficiently expressed".

 In the section "Organising gene annotations" I think it's good the authors point out to check for duplicated genes, however in this case the duplication appears to be due to extracting the TXCHROM column (as some genes are reported as being present on more than one chromosome) but the TXCHROM information is not used in this workflow and if the TXCHROM column is omitted then there are no duplicates so it might be worth mentioning that.

 With this bit "Differential expression analyses look at gene expression differences between conditions, rather than comparing expression across multiple genes or drawing conclusions on absolute levels of expression. In other words, gene lengths remain constant for comparisons of interest and any observed differences are a result of changes in condition rather than changes in gene length." Wouldn't one caveat to this be if there was a significant change in the length of the isoform(s) expressed from a gene (e.g. from expression of a long isoform to a short isoform) as then the assumption of no change in gene length would no longer be valid.

 In the online version Fig.7 is in the middle of a code block could it be moved below.

 Figs 5-8 have bold headings but Figs 1-4 don't is that an error.

 Could perhaps modify title to reflect the order in which the tools are used - edger, limma & Glimma.

 It would be nice for consistency if the colours in the Glimma interactive MDS plots matched the colours used in the static MDS plots in Fig 3.

I have read this submission. I believe that I have an appropriate level of expertise to confirm that it is of an acceptable scientific standard.

# Response

**Published:** 2016-07-06

**Authors:** Maria A. Doyle

**Affiliations:** Peter MacCallum Cancer Centre, Australia

I've just requested that the status of this article be changed to Approved as the code works fine with the development release of Bioconductor 3.4 but could it be made clearer in the article that 3.4 must be used for the code to work as shown.

# Response

**Published:** 2016-11-30

**Authors:** Charity Law

**Affiliations:** The Walter and Eliza Hall Institute of Medical Research, Australia

Dear Maria, we have made changes according to your suggestions, including a change in the section title to "Removing genes that are lowly expressed", and we have added comments to point out that our analysis assumes no differential isoform usage. We have also simplified our example in the "Organising gene annotations" section.

 Thanks for pointing out the inconsistencies in the figure captions. They have now been addressed.

 Indeed, it would be nice to make the colours consistent between the interactive and static MDS plots. We like the colours used in the static plot and can have the interactive version of the plot updated once color specification becomes available to the MDS plotting function in Glimma.

# Peer review: Referee response for version 1

**DOI:** 10.5256/f1000research.9688.r14436

**Published:** 2016-06-23

**Authors:** James W. MacDonald

**Affiliations:** 1 Department of Environmental and Occupational Health Sciences, University of Washington, Seattle, WA, USA

In this manuscript the authors perform a step-by-step analysis of a public RNA-Seq data set, using the Bioconductor packages edgeR, limma, and Glimma. The analysis proceeds through four stages (data preparation, preprocessing, univariate analyses, and gene set testing), with each stage broken up into several discrete steps. Each step is clearly explained and is accompanied by R code and output, so the reader can follow along if desired.

 The target audience for this paper appears to be someone with passing familiarity with both R and statistics, without requiring expertise in either. Most of the code should be easy to understand, and any complex sections are clearly explained in the text. Similarly, the authors outline the analytic choices they make (e.g., filtering out unexpressed genes, specifying model coefficients, etc) at a more accessible level.

 While both edgeR and limma are well known, popular Bioconductor packages, Glimma is a new package that was released in April 2016. This package uses the d3.js JavaScript library to generate interactive HTML documents that can be viewed locally, rather than needing to be accessed from a server running R (as, say a shiny app requires). This is an exciting development, and is unfortunately not as compelling as it could be, if the Glimma plots were part of the HTML version of the manuscript rather than provided as links.

 This is a well written paper, and is a useful contribution to the literature; while each package has either an extensive user's guide or vignette, by necessity these documents pertain only to the package at hand. Most RNA-Seq analyses require a combination of multiple packages to complete, and this paper provides a clear example.

**Major comments**

 None. 

**Minor comments**

 In the section 'Organising gene annotations', the code used to subset the one-to-many mappings is needlessly complex. Simply doing something like

 genes <- genes[!duplicated(genes[,1]),]

 will accomplish the same thing, in a more straightforward way.

 In the section 'Removing heteroscedasticity from count data', the authors state:

*"When operating on a DGEList-object, voom converts raw counts to log-CPM values by automatically extracting library sizes and normalisation factors stored in the object. For a matrix of counts, the method of normalisation can be specified within voom using the normalize.method (by default no normalisation is performed)."*

 This is confusing; the authors already showed in an earlier section ('Normalising gene expression distributions') that converting to log-CPM using TMM normalization factors will do a shift-normalization (which is what voom will do in this instance). The normalize.method argument to voom specifies **additional** normalization methods that can be applied to the matrix of TMM normalized log-CPM values.

I have read this submission. I believe that I have an appropriate level of expertise to confirm that it is of an acceptable scientific standard.

# Response

**Published:** 2016-11-30

**Authors:** Charity Law

**Affiliations:** The Walter and Eliza Hall Institute of Medical Research, Australia

Dear James, thank-you for your comments. We have considered your suggestions and made changes to simplify the section on ‘Organising gene annotations’ and to clarify that voom offers additional normalisation ontop of the TMM-normsalisation that is carried out.
